# Granular estimation of user cognitive workload using multi-modal physiological sensors

**DOI:** 10.3389/fnrgo.2024.1292627

**Published:** 2024-02-27

**Authors:** Jingkun Wang, Christopher Stevens, Winston Bennett, Denny Yu

**Affiliations:** ^1^School of Industrial Engineering, Purdue University, West Lafayette, IN, United States; ^2^Air Force Research Laboratory, Wright-Patterson AFB, Dayton, OH, United States

**Keywords:** mental workload, mental workload modeling, physiological sensors, teleoperation task, multiple mental workload level

## Abstract

Mental workload (MWL) is a crucial area of study due to its significant influence on task performance and potential for significant operator error. However, measuring MWL presents challenges, as it is a multi-dimensional construct. Previous research on MWL models has focused on differentiating between two to three levels. Nonetheless, tasks can vary widely in their complexity, and little is known about how subtle variations in task difficulty influence workload indicators. To address this, we conducted an experiment inducing MWL in up to 5 levels, hypothesizing that our multi-modal metrics would be able to distinguish between each MWL stage. We measured the induced workload using task performance, subjective assessment, and physiological metrics. Our simulated task was designed to induce diverse MWL degrees, including five different math and three different verbal tiers. Our findings indicate that all investigated metrics successfully differentiated between various MWL levels induced by different tiers of math problems. Notably, performance metrics emerged as the most effective assessment, being the only metric capable of distinguishing all the levels. Some limitations were observed in the granularity of subjective and physiological metrics. Specifically, the subjective overall mental workload couldn't distinguish lower levels of workload, while all physiological metrics could detect a shift from lower to higher levels, but did not distinguish between workload tiers at the higher or lower ends of the scale (e.g., between the easy and the easy-medium tiers). Despite these limitations, each pair of levels was effectively differentiated by one or more metrics. This suggests a promising avenue for future research, exploring the integration or combination of multiple metrics. The findings suggest that subtle differences in workload levels may be distinguishable using combinations of subjective and physiological metrics.

## 1 Introduction

Teleoperation tasks are often high-stakes, requiring operators to process multi-dimensional, and multi-channel information (Wickens, 2008; Hockey et al., 2009). Studies have shown that teleoperation interfaces and technology may increase complexity and workload for operators compared to non-teleoperated tasks (Dadashi et al., 2013; Yu et al., 2021; Monfared et al., 2022), such as robotic surgery vs. traditional open surgery. When these job demands exceed human mental capabilities, it can degrade the capabilities of teleoperation personnel and the success of the operation. Thus, continuous and reliable assessment of operators' MWL across high and low ranges is essential, not only to protect operators but also to maximize teleoperation task performance.

While there is no universally accepted definition for MWL (Schlegel, 1993; Cain, 2007; Young et al., 2015), Longo et al. (2022) identified that main concepts (such as operator, primary task, secondary task, environment, situation, time, performance, system, and decision-making), along with sub-concepts (including attributes, demands, resources, effort, attention, working memory, and characteristics), and their interactions, all contribute to the definitions of Mental Workload (MWL). Furthermore, multiple theories propose a resource framework to describe MWL, which characterizes MWL as the extent to which a finite set of cognitive resources is taxed by a given set of task demands (Young and Stanton, 2004; Wickens, 2008; Salvucci and Taatgen, 2010). If task demands are too high with respect to an operator's available capacity, the operator's performance may decline or the operator could become easily distracted, tense, and frustrated (Norman and Draper, 1986; Monk et al., 2002; Galy et al., 2012; Pourteimour et al., 2021). Conversely, excessively low MWL may lead to operator inattentiveness and decreased performance because of the effort associated with sustained attention (Kantowitz and Casper, 1988; Hart and Wickens, 1990). Therefore, whether high or low, MWL can impact task success and the operator's state. For this reason, rapid detection of variations in workload state can be important for preventing errors.

As MWL is a high-level cognitive function that changes dynamically, reliable measurement of MWL is challenging. Three categories of techniques have been widely accepted to assess MWL: subjective measurement, physiological measurement, and performance (Meshkati et al., 1995). Rubio et al. (2004) detailed a list of common subjective questionnaires that assesses MWL, and Hicks and Wierwille (1979) found there were significant differences between workload conditions on subjective and performance measures. A systematic review conducted by Charles and Nixon (2019) detailed 58 journal articles and demonstrated the empirical basis for using physiological sensors in quantifying MWL across a variety of domains. Studies in teleoperation tasks, such as robotics and drone operations, have also shown that physiological sensors can assess workload in those domains (Dias et al., 2018; Yu et al., 2019; Zhou et al., 2020). However, previous studies have primarily focused on the sensors' effectiveness in detecting large changes or classifying high vs. low MWL, limiting the granularity of existing models (their ability to detect fine, multi-level changes in workload).

Each measurement method has its advantages and drawbacks. While subjective measurements can be easier and inexpensive, the result of subjective ratings can be impacted by respondent characteristics like bias, response sets, mistakes, and attitudes (Dyer et al., 1976). Primary task performance-based information has the ability to distinguish between individual differences when competing for resources (Longo, 2015), but different demand levels may be associated with the same level of performance. Online performance metrics may also not be available. Physiological measures provide relatively unobtrusive measurement and lack of subjectivity. However, it requires specific measurement equipment, and data quality can be compromised by motion and other artifacts (Dirican and Göktürk, 2011). Integrating all three methods is crucial for a comprehensive understanding of MWL.

In past studies, researchers investigated whether MWL measurement(s) can differentiate between low, medium, and high MWL levels (May et al., 1990; Miller et al., 2011) and developed models to distinguish between two or three levels (Ding et al., 2020; Zhou et al., 2022). For jobs that are susceptible to a wide range of more granular changes, such as high-stakes tasks and the environments in search and rescue (SAR), these 2-levels (low vs. high) or 3-levels models may be insufficient for personalized and more targeted design of mechanisms for enhancing operator performance across a range of workloads. More granular models can potentially track gradual changes in operator MWL before they reach excessively high or low levels. These sensitive models can also provide more targeted interventions and potentially improve the usability of operators' collaboration with SAR robotic systems.

The objective of the proposed study is to determine the effectiveness of subjective questionnaire, task performance, and physiological sensors in modeling gradual changes in MWL. To achieve our goal, a teleoperated snow arctic SAR gaming simulation was adapted to systematically modulate user workloads at multiple levels. SAR tasks were selected for our testbed as human operators often experience stress, cognitive and physical fatigue, and disorientation during critical SAR operations. These factors can lower alertness, impair memory and focus, and result in a loss of situation awareness (Casper et al., 2000; Murphy, 2001, 2007; DeJong et al., 2004; Zhao et al., 2017).

## 2 Material and methods

This research adhered to the American Psychological Association Code of Ethics and received approval from the Institutional Review Board at Purdue University (IRB No.: IRB-2021-1152). Informed consent was obtained from all participants.

### 2.1 Participants

Twenty-three participants (12 males and 11 females) were recruited from the university population. The exclusion criteria for this study were as follows: (1) age younger than 18 years old and (2) an inability to play video games without discomfort. Before conducting the experiment, all participants provided informed consent.

### 2.2 Experimental design

In this study, workload is induced experimentally by varying task demands. In addressing operator capacity, attempts were undertaken to minimize individual differences by (1) considering the participant as a random factor in our model and (2) augmenting the sample size within our capacity. Participant backgrounds, skills, and learning experiences were surveyed prior to the study.

To distinguish different MWL levels in arithmetic problems, mathematical operations were decomposed into steps. Steps were defined as basic (single-digit) arithmetic calculations. Each step involved retrieving the solution from long-term memory (Ashcraft, 1992). However, multi-digits operations and multi-step problems are more complex as they require working memory (WM) to store addends and interim results (Hitch, 1978). Increasing the number of addends and interim values that need to be stored demands more WM resources, leading to a higher MWL. Additionally, larger numbers with more digits tend to require more retrievals of number facts from memory to compute intermediate sums, resulting in increased MWL (Ryu and Myung, 2005). Following this rule, we defined five different math demand levels, referred to as “question demand levels” throughout this manuscript. These levels include easy (e.g., 8^*^2 + 5 or 4^*^6 – 7); easy-medium (e.g., 75 + 48); medium (e.g., 5^*^5 + 3^*^6 or 8^*^8 + 9^*^2); medium-hard (e.g., 14^*^3 – 16); and hard [e.g., 2^*^(26 + 48 – 14 – 32)].

The cognitive basis for varying MWL levels with analogy questions differed from the working memory demand of arithmetic problems. In this case, the difficulty comes from the complexity of inference rules associated with the question and the availability of verbal knowledge associated with the terms (Bejar et al., 1987). Problem difficulty was defined normatively, drawing from data obtained from a sample of SAT I practice tests. Analogy questions were selected from a chart of example SAT questions with corresponding categories based on a reference group of high school seniors planning to attend college.^1^ The verbal questions in these practice tests were categorized into 5 demand levels. However, we retained only 3 levels of demand (MWL levels) in the study, as our pilot testing revealed that the demands of analogy tasks were challenging and not consistently clear among individuals. All verbal analogy questions were presented in a multiple-choice format.

This study employed an incomplete block design. Initially, a complete block design was planned, with each participant expected to answer 8 (5 math and 3 verbal) different levels of the questions for each round, two rounds in total. However, due to a game programming error, each participant only answered 7 questions per round, resulting in an incomplete block design. Nevertheless, since our study was comprised of two rounds per subject, all but one subject experienced all 8 treatment levels at least once. Additionally, we conducted separate analyses for math and verbal questions. Specifically, 5 different math demand levels (3 different analogy demand levels) were employed as the treatment factors in math (verbal) models.

### 2.3 Simulation description

A series of gaming simulations developed by Air Force Research Laboratory (AFRL) Gaming Research Integration for Learning Laboratory (GRILL) was employed in this study. The game simulated Antarctic Search and Rescue operations with a teleoperated snowcat (Coovert et al., 2017a,b). In this simulation scenario, two scientists embarked on a helicopter mission to gather research data. However, the helicopter suddenly malfunctioned and crashed during an emergency landing on snowy terrain.

Participants were tasked with operating a snowcat to perform a search and rescue operation, aiming to locate parts at various milestones to repair the helicopter. As participants advanced in the simulation and answered questions correctly, they could obtain these parts at each milestone. To achieve this, participants teleoperated a snowcat across a vast arctic terrain, with a total of 16 milestones to be reached within a 15-min timeframe.

**Figure 1:**
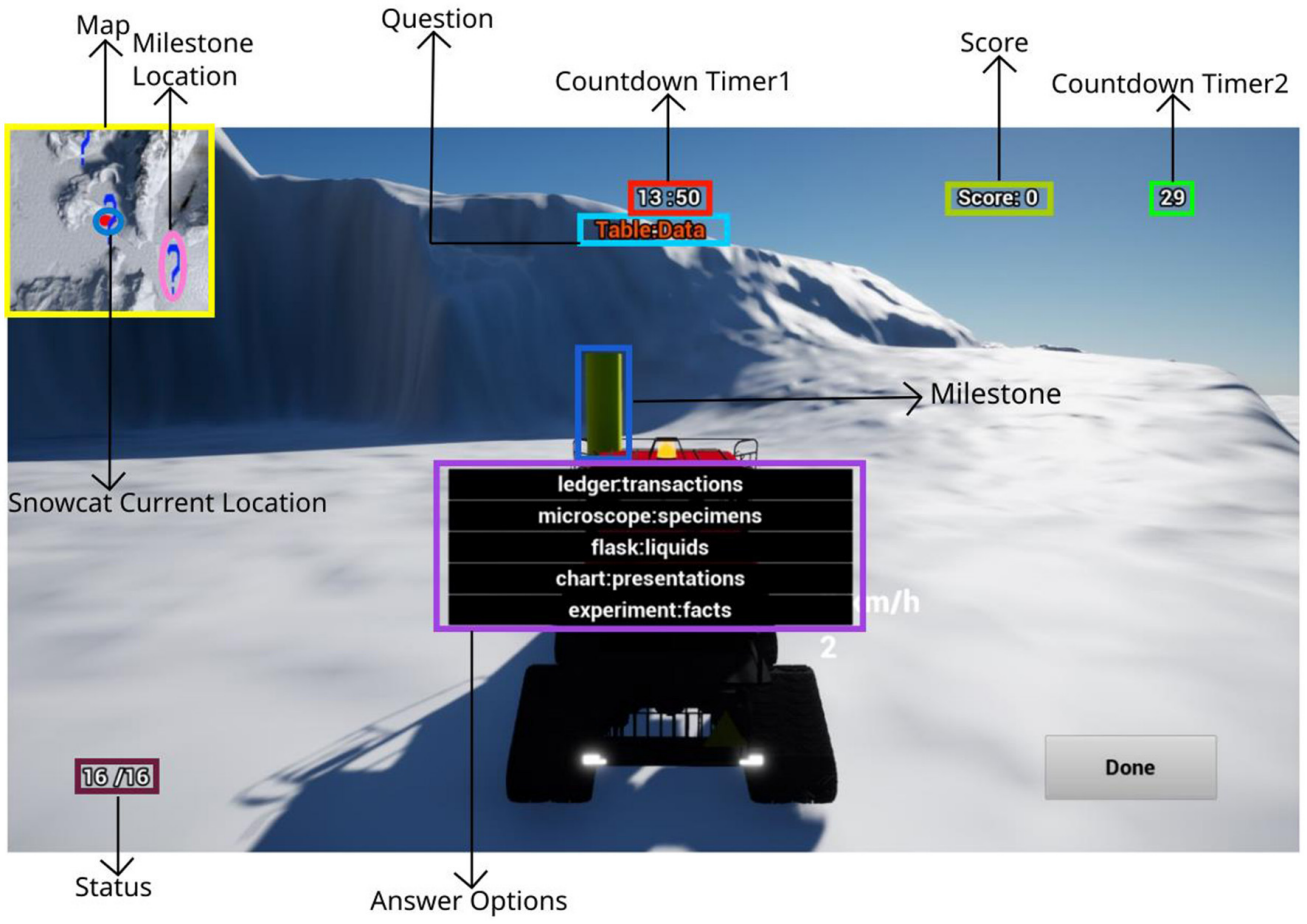
Interface of the snowcat simulation. The area map in the upper-left corner indicated the milestone locations and the current snowcat vehicle location. The countdown timer 1 indicated how much time is left out of 15 min. Milestone status in the lower-left corner indicated how many milestones were found.

At each milestone, participants encountered an arithmetic or analogy question with a randomized MWL demand, as depicted in Figure 1. Correct answers caused the total score to go up, while the wrong answer caused the total score to go down. Only when a correct answer was provided did a new question appear, maintaining the same demand level and question type at that milestone. A wrong answer required participants to answer the question again until they answered it correctly. Participants were given 30 seconds to answer as many questions as possible at each milestone, with Countdown Timer 2 (Figure 1) displaying the time remaining.

To ensure that participants engaged with at least one set of seven demand levels of problems (randomly selected from five levels of arithmetic and three levels of analogy), the demand levels and/or problem types for the first seven milestones differed. Subsequently, problems were fully randomized and excluded from further analysis.

### 2.4 Experimental procedure

**Figure 2:**
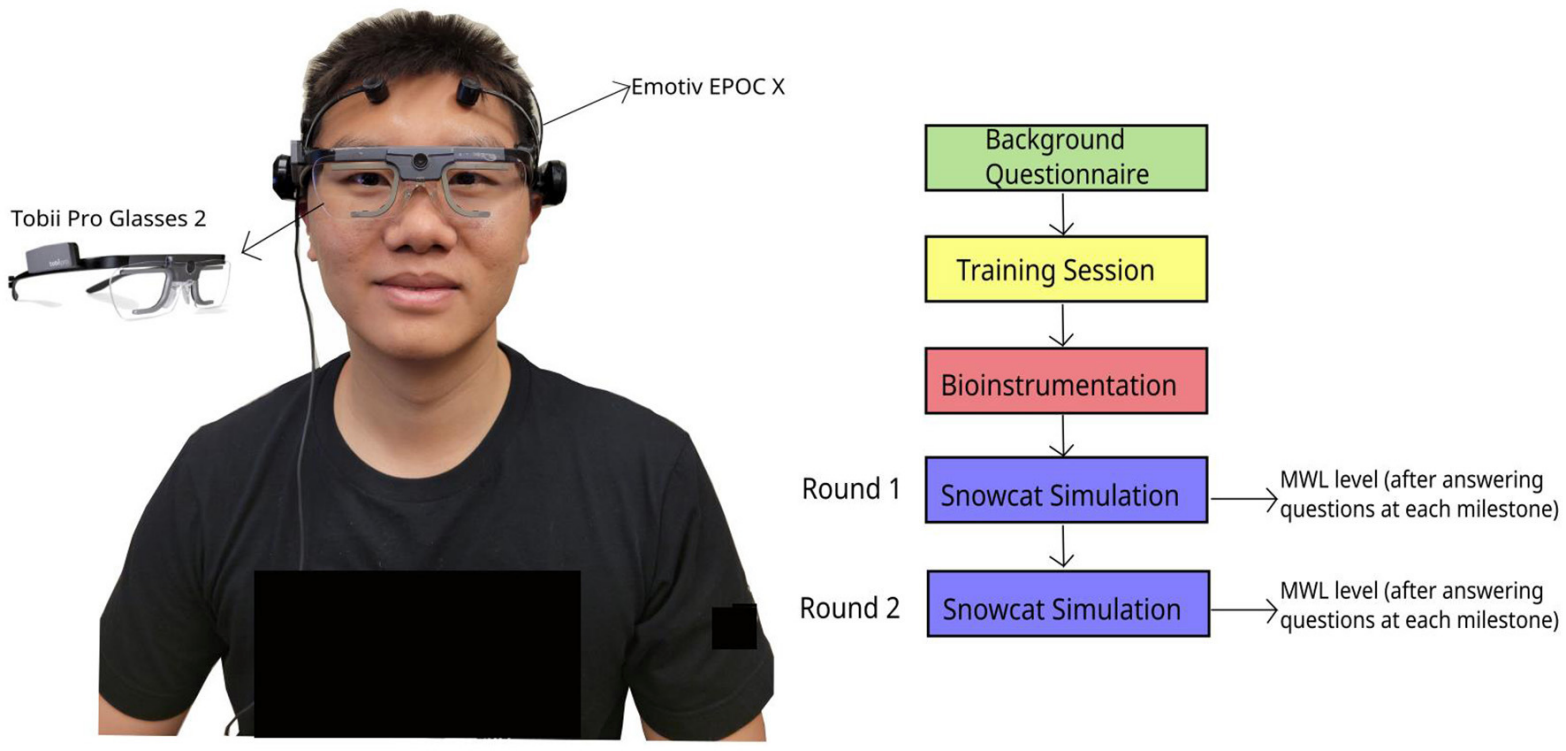
Sensors (EEG and Eye Tracker) and experimental procedure.

The experimental procedure is shown in Figure 2. Prior to commencing the experiment, participants were required to complete a background questionnaire, which collected general information, including sex, age, and whether they were native English speakers (see Appendix A). We included the native English speaker question based on insights from pilot studies, as performance on analogy problems might differ between native and non-native English speakers.

Following the background questionnaire, participants were introduced to a single 20-step bipolar scaled Overall Workload (OW) questionnaire (Hill et al., 1992; Miller, 2001). Then, we asked participants to self-report their MWL levels on a scale ranging from 1 to 21. The OW score was then calculated as *OW* = (*reported scale* − 1)^*^5, resulting in a score between 0 and 100 (Hill et al., 1992).

A training session was conducted for all participants. During this session, research personnel explained the objectives and interfaces of the simulation and allowed participants to practice the snowcat simulation. Following the training session, participants were equipped with the Emotiv EPOC X and Tobii Pro Glasses 2. Eye tracker calibration was performed to optimize the eye tracking algorithm.^2^ Each participant engaged in two rounds of the snowcat simulation, each round lasted 15 min. While the snowcat simulation itself remained consistent across both rounds, the milestone questions differed due to randomization. After completing questions at each milestone within the snowcat simulation, participants reported their OW for that specific milestone.

### 2.5 Subjective data and performance metrics

Participants self-reported OW questionnaire was recorded after they completed questions at a milestone. The OW score ranged from 0 (indicating a very light workload) to 100 (representing a very high workload) (Hill et al., 1992).

Task performance metrics for the snowcat simulation were defined as the total number of questions answered and the percentage of correct questions answered.

### 2.6 Physiological data analysis

#### 2.6.1 Eye tracking data analysis

Tobii Pro Glasses 2 served as the eye-tracking device in this study. Eye-tracking data was recorded using the Tobii Pro Controller, and ocular measurements were extracted using the Tobii Pro Lab. The I-VT attention filter was set as the default value of 100,^3^ following the manufacturer's recommendations (Bahill, 1975; Bahill et al., 1975; Collewijn et al., 1988).

Previous research has demonstrated correlations between multiple metrics and MWL, including fixation duration, the number of fixations, saccadic velocity, saccade amplitude, maximum saccade duration, number of saccades, and pupil size (Ahmadi et al., 2022). Consequently, metrics associated with these parameters were extracted for each 30-s milestone.

#### 2.6.2 Electroencephalography data analysis

We used Emotiv EPOC X as our EEG device. We determined the EEG metrics to be the extracted alpha, beta, and theta bandpowers from the 14 EEG channels (AF3, F7, F3, FC5, T7, P7, O1, O2, P8, T8, FC6, F4, F8, and AF4). Following the export of raw data from Emotiv Pro, we pre-processed the EEG data using EEGLab. Event markers were imported to identify the time periods corresponding to each demand level. For each time period, the following steps were completed. (1) a high pass filter at 0.5 Hz was applied; (2) the clean_rawdata algorithm was applied to automatically reject bad segments and channels (Delorme and Makeig, 2004); (3) researchers manually reviewed the data to identify and reject any remaining bad segments and channels; (4) the runica algorithm was utilized to decompose data using independent components analysis (ICA) (Brunner et al., 2013); (5) the ICLabel algorithm was employed to classify each ICA component into categories such as brain, eye, muscle, heart rate, line noise, or others, quantifying their probability on a percentage scale (Pion-Tonachini et al., 2019); (6) ICA components with a probability of belonging to the brain of <0.4 were rejected; and (7) MATLAB scripts were used to calculate alpha, beta, and theta value for each channel based on the method provided by previous study (Wang et al., 2015).

### 2.7 Statistical analysis

Nested models were used as round was nested within participant. Specifically, the fixed effect consisted of 5 different demand levels for math and 3 different demand levels for verbal models. The random effects include the participant and the round completed by each participant, with MWL measurement metrics serving as the responses. Separate models were built for each subjective, task performance, eye tracking, and EEG metrics. Each model expression was: *Y*_*ijkt*_ = μ + α_*i*_ + β_*j*(*k*)_ + ϵ_*ijkt*_, where *Y*_*ijkt*_ represents the metric measurement on the t^th^ observation on demand Level i, observed in participant k and round j; μ denotes the constant (or the intercept of the model); α_*i*_ signifies the effect of the demand Level i; β_*j*(*k*)_ represents the nested effect of the j^th^ round of the k^th^ participant; and ϵ_*ijkt*_ represents the associated random error.

We initiated the analysis by checking statistical model assumptions, including the examination of outliers, independence, constant variance, and normality. Outliers were identified as values exceeding $Q_{3}+1.5^{*}$IQR or falling below $Q_{1}-1.5^{*}$IQR, where *Q*_3_ and *Q*_1_ represent the third and first quartiles; and the interquartile range (IQR) is defined as *Q*_3_ − *Q*_1_. After quantitatively identifying outliers, each data point was visually inspected. It was determined that outliers occurred due to some participants employing different strategies when solving math problems. For example, one participant typed partial sums as typed answers in the answer option box when responding to math questions (if the question is 8^*^8+9^*^2, he would type 64 and 18). We opted to remove the milestone data points when they were identified as outliers, typically totaling 2 to 3 data points for each metric across the entire dataset. Next, studentized residual plots were generated to assess the independence and constant variance. Lastly, we produced normal residual plots (standardized residuals plotted against their normal scores). If non-normality or non-constant variables were detected, we applied Box-Cox transformations. As the relationship between performance metrics and the demand levels is unknown, we also attempted to apply square root, exponential, and logarithmic transformations.

After confirming the model assumptions were met, we utilized PROC MIXED^4^ to conduct nested model analysis, considering both random and fixed factors. For variables with significant fixed effects, pairwise comparisons were conducted with the Tukey correction using the LSMEANS command under PROC MIXED. *P*-values of the differences of the least squares indicated the various levels of significance for each metric.

## 3 Results

### 3.1 Background questionnaire result

Twenty-three participants were recruited. Only twenty participants (11 males and 9 females) data were collected and analyzed in this study. EEG failed to work for three of the participants. The means (standard deviations) of the age of male and female participants were 28.18 (12.42) and 24.89 (5.98), respectively. The means (standard deviations) for familiarity with, frequency of, and confidence in playing video games were 3.00 (1.45), 2.32 (1.36), and 3.09 (1.23), respectively.

### 3.2 Task performance result

Both the number of questions answered (*p* < 0.0001) and the percentage of correct answers (*p* < 0.0001) decreased when math demands increased (Table 1). For the verbal problems, the number of questions answered was similar between demand levels, but the percentage of correct answers decreased (*p* = 0.0010) when verbal question demands increased.

**Table 1:** Average and standard deviation values of two performance metrics for each demand level.

**Demand level**	**# of questions answered (average)**	**# of questions answered (Std.)**	**Average % of correct answer (average)**	**Average % of correct answer (Std.)**
Math 1	7.56	3.65	88.86	19.51
Math 2	5.88	2.34	89.31	12.84
Math 3	3.40	2.14	74.65	36.65
Math 4	2.42	1.30	63.89	41.13
Math 5	1.15	1.21	37.84	46.43
Verbal 1	2.85	1.37	54.35	33.48
Verbal 2	2.91	1.20	46.77	33.79
Verbal 3	2.63	1.50	24.12	34.72

**Figure 3:**
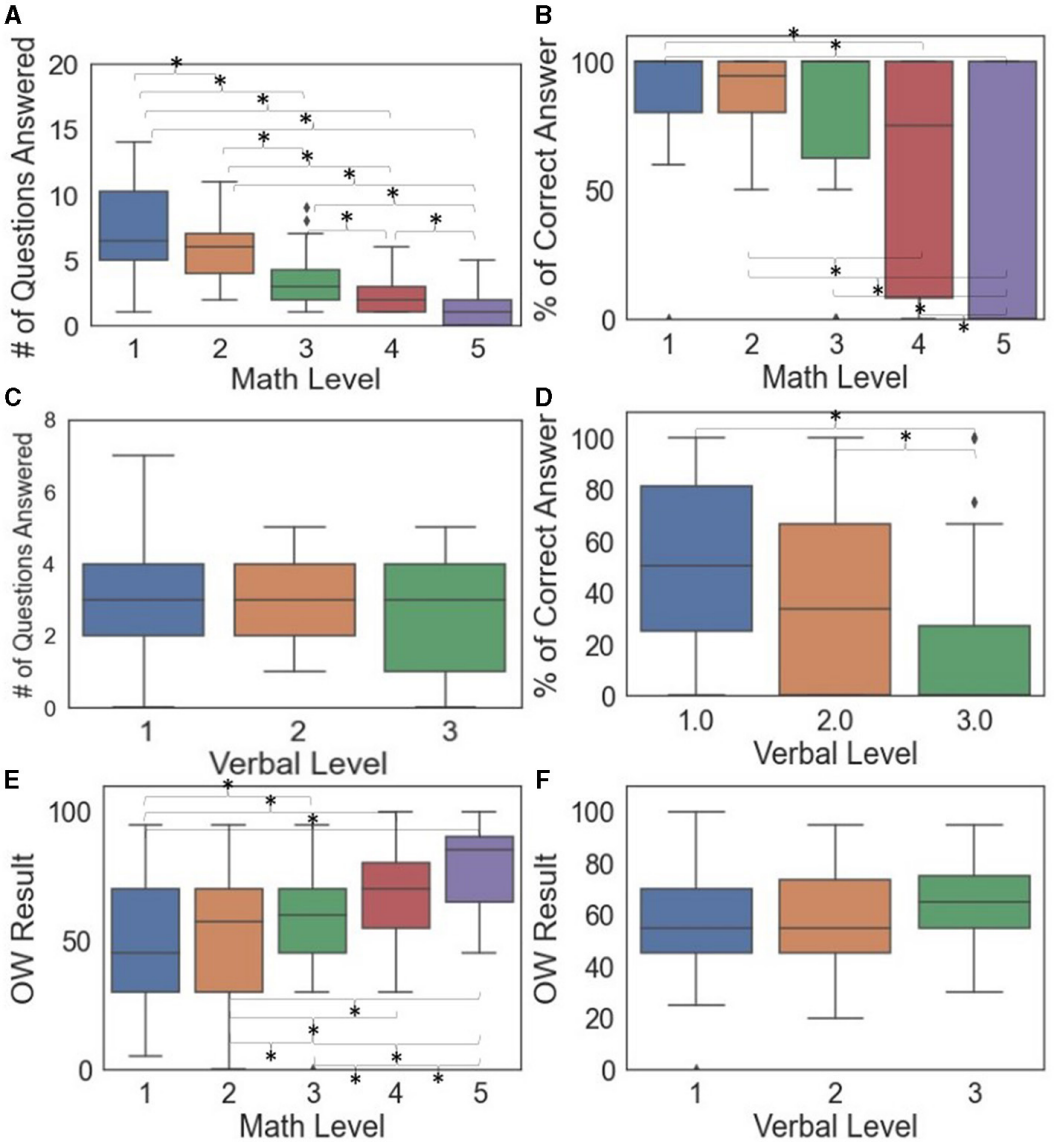
Box plots of task performance **(A–D)** and subjective questionnaire **(E, F)** by demand levels where significant differences between pairs indicated by * when *p* < 0.05.

Figures 3A–D shows the box plots and pairwise comparisons between demand levels and task performance. The number of math questions answered differed between all pairs of math demand levels (Figure 3A). The percentage of correct answers was less sensitive to math demand levels, with only select pairs significantly different (1&4, 1&5, 2&4, 2&5, 3&5, and 4&5). The percentage of correct answers for verbal problems distinguished verbal Levels 1&3 and 2&3, but not 1&2 (Figure 3D). Appendixes B.I, B.II show more details of the significant task performance pairwise comparison results of math-based and verbal-based models.

The transformed performance metrics did not contribute significantly to improving the model fit, except for the square root transformation of the percentage of correct answers for the math levels. This transformation indeed facilitated the significance of one additional pair (2&3). Appendixes B.I.2-4, B.II.2-3 provide more details of the significant pairwise comparisons of task performance for math-based and verbal-based models after transformations.

### 3.3 Subjective questionnaire result

Self-reported OW score (Figure 3E, F) increased when the demand levels increased for math problems (*p* < 0.0001) but not verbal problems (*p* = 0.2752). From pairwise comparisons (Figure 3E), all pairs of math demands were significantly different except for 1&2. Appendix B.III shows additional details of the pairwise comparison results.

### 3.4 Eye tracking

The results of the eye-tracking models are shown in Table 2. If Box-Cox transformation was applied due to non-normality and/or a non-constant variable, the corresponding type of the transformation is also shown in Table 2. For math-based models, no metric needed to be transformed. For verbal-based models, all metrics except total fixation duration, number of saccades, and total amplitude of saccades were transformed.

**Table 2:** Transformation and ANOVA result for each eye tracking metric.

**Extracted eye tracking metrics**	**Math-based models transformation**	***P*-value of the math fixed factor**	**Verbal-based models transformation**	***P*-value of the verbal fixed factor**
Total fixation duration	-	0.0029^*^	-	0.6381
Average fixation duration	-	<0.0001^*^	$\frac{1}{\sqrt{Y}}$	0.2381
Number of fixations	-	<0.0001^*^	log(Y)	0.1992
Average fixation pupil diameter	-	0.3315	*Y* ^−2^	0.3579
Number of saccades	-	<0.0001^*^	-	0.1219
Average peak velocity of saccade	-	0.1503	*Y* ^−2^	0.0399
Average amplitude of saccades	-	0.0053^*^	$\frac{1}{\sqrt{Y}}$	0.2271
Total amplitude of saccades	-	<0.0001^*^	-	0.3417

Significance marked as ^*^ when *p* < 0.05.

**Figure 4:**
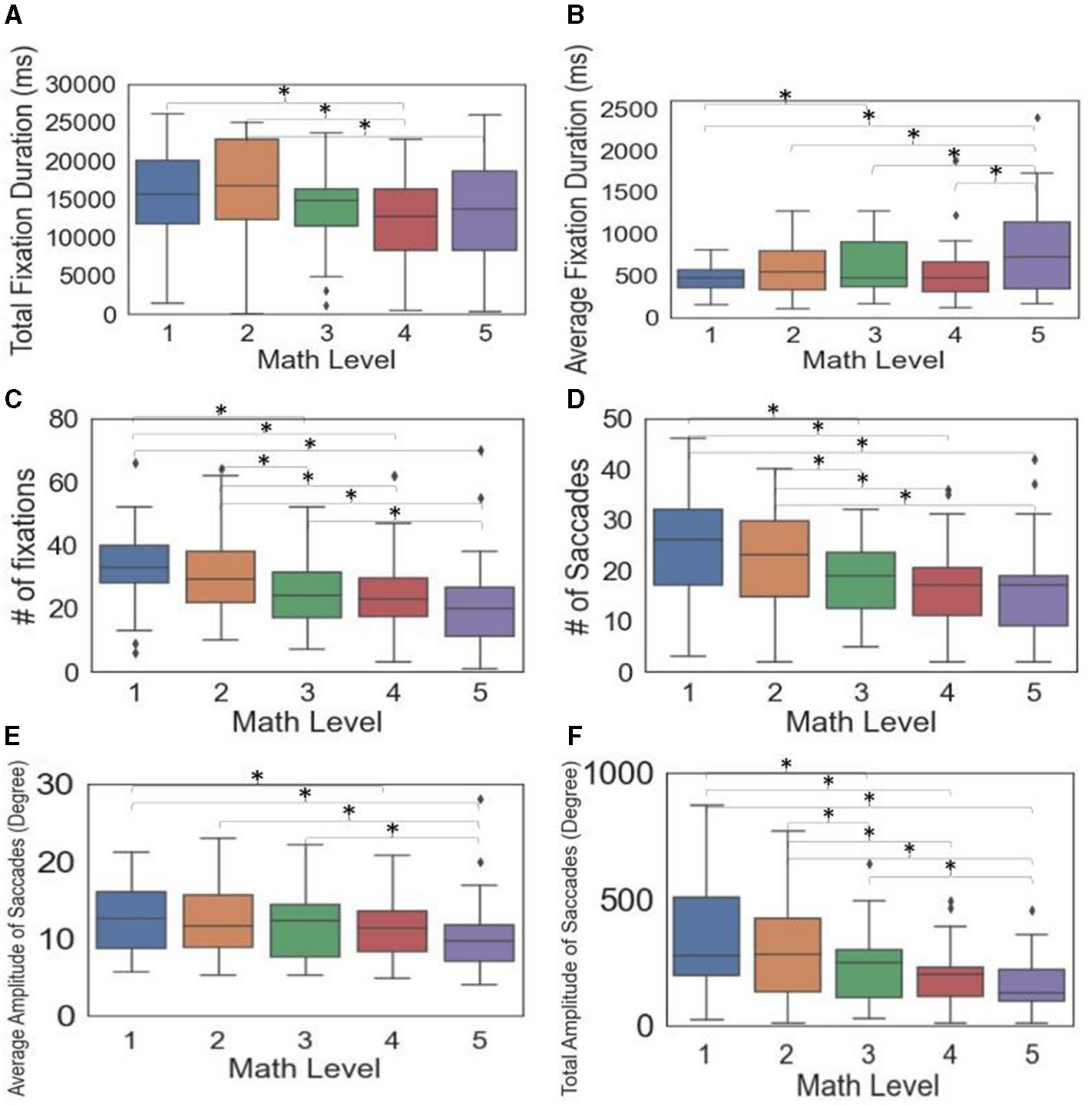
Box plots and significance among math levels for eye tracking metrics **(A–F)** (Significant differences between pairs indicated by * when *p* < 0005).

Testing for multiple comparisons among the significant metrics (Table 2) showed that some, but not all, demand level pairs differed in math-based models (Figure 4). The metrics, including total fixation duration, number of fixations, number of saccades, average amplitude of saccades, and total amplitude of saccades, generally decreased with increasing demand levels (Figure 4). In contrast, the average fixation duration tended to increase with higher demand levels. Specifically, at Level 1, the duration was 148 ms and 342 ms shorter than at Levels 3 and 5, respectively, while Levels 2–4 were 195–261 ms shorter than Level 5. For additional details on the pairwise comparison results, see Appendix B.IV.

**Figure 5:**
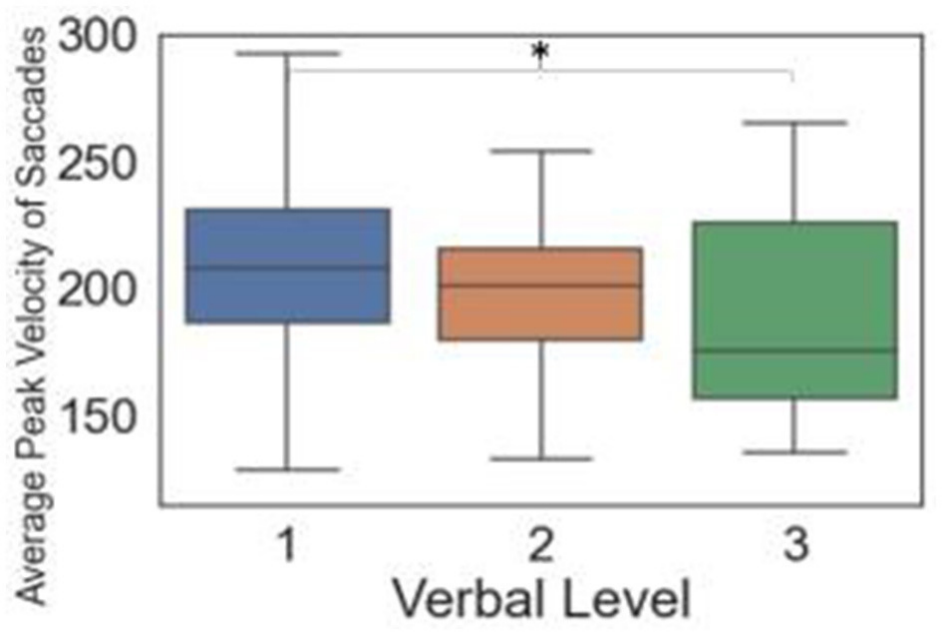
Box plots and significance among verbal levels for eye tracking metrics (Significant differences between pairs indicated by * when *p* < 0.05).

For verbal questions, only the average peak velocity of saccades was significant. Specifically, verbal Level 3 was significantly lower than Level 1 (Figure 5). Appendix B.V shows additional details on the pairwise comparison results.

### 3.5 EEG

For math-based models, the following EEG metrics distinguished MWL levels: log(alpha) of channel F8 (*p* = 0.0056); log(beta) of channel T7 (*p* = 0.0496) and channel F8 (*p* = 0.0031); log(theta) of channel T7 (*p* = 0.0483), T8 (*p* = 0.0374), and F8 (*p* = 0.0049).

**Figure 6:**
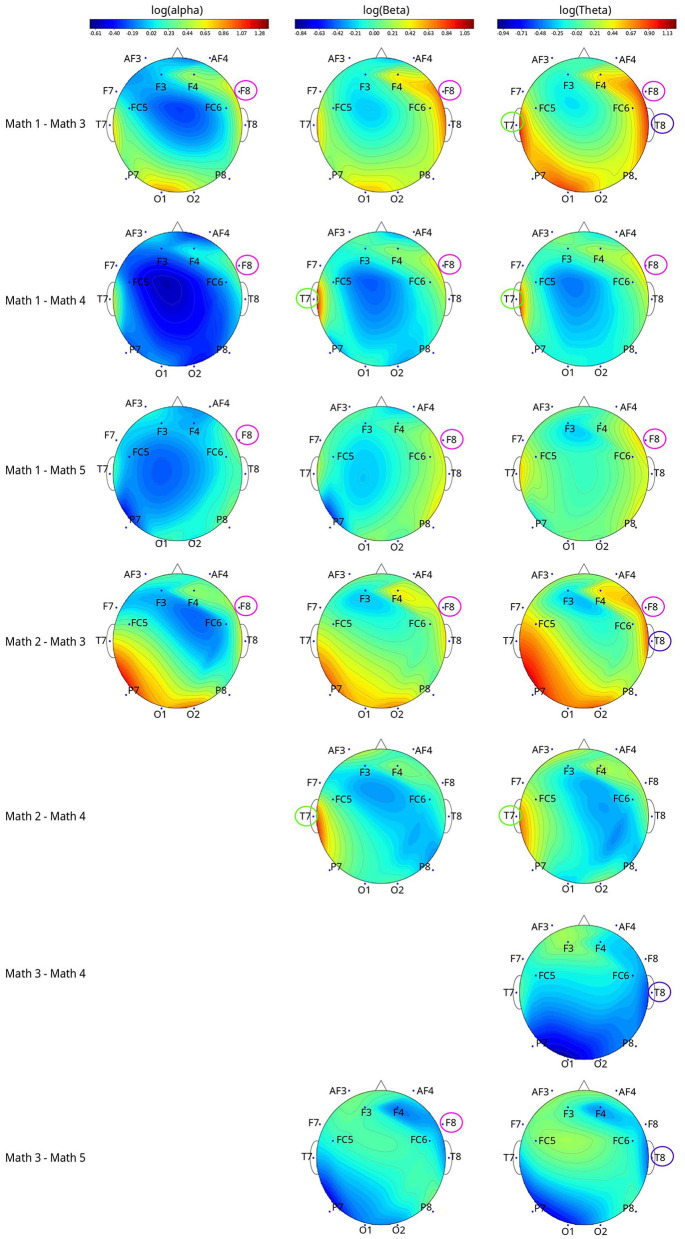
The topographical plots of the significant metric (after applying log transformation) among different math levels with channels that differed between pairwise comparisons circled. The topographical plots were made based on the value difference between two levels of log(metric).

None of the EEG metrics can distinguish all pairs of math levels (Figure 6). For log(alpha) of F8, log(beta) of T7, log(theta) of T7, and F8, the bandpowers showed inverse relationships with the demand levels. For example, log(alpha) of F8 metric math Level 1 was 0.89, 0.48, and 0.45 higher than Levels 3, 4, and 5; Level 2 was 0.61 higher than Level 3. Note that the log transformations and original metrics have positive relationships, the same tendency could be applied to the original alpha, beta, and theta metrics in the above interpretations.

Bandpowers of log(beta) of F8 and log(theta) of T8 metrics tended to decrease and then increase when the demand levels increased. Appendix B.VI shows additional details on the math-based significant EEG metrics pairwise comparison results.

**Figure 7:**
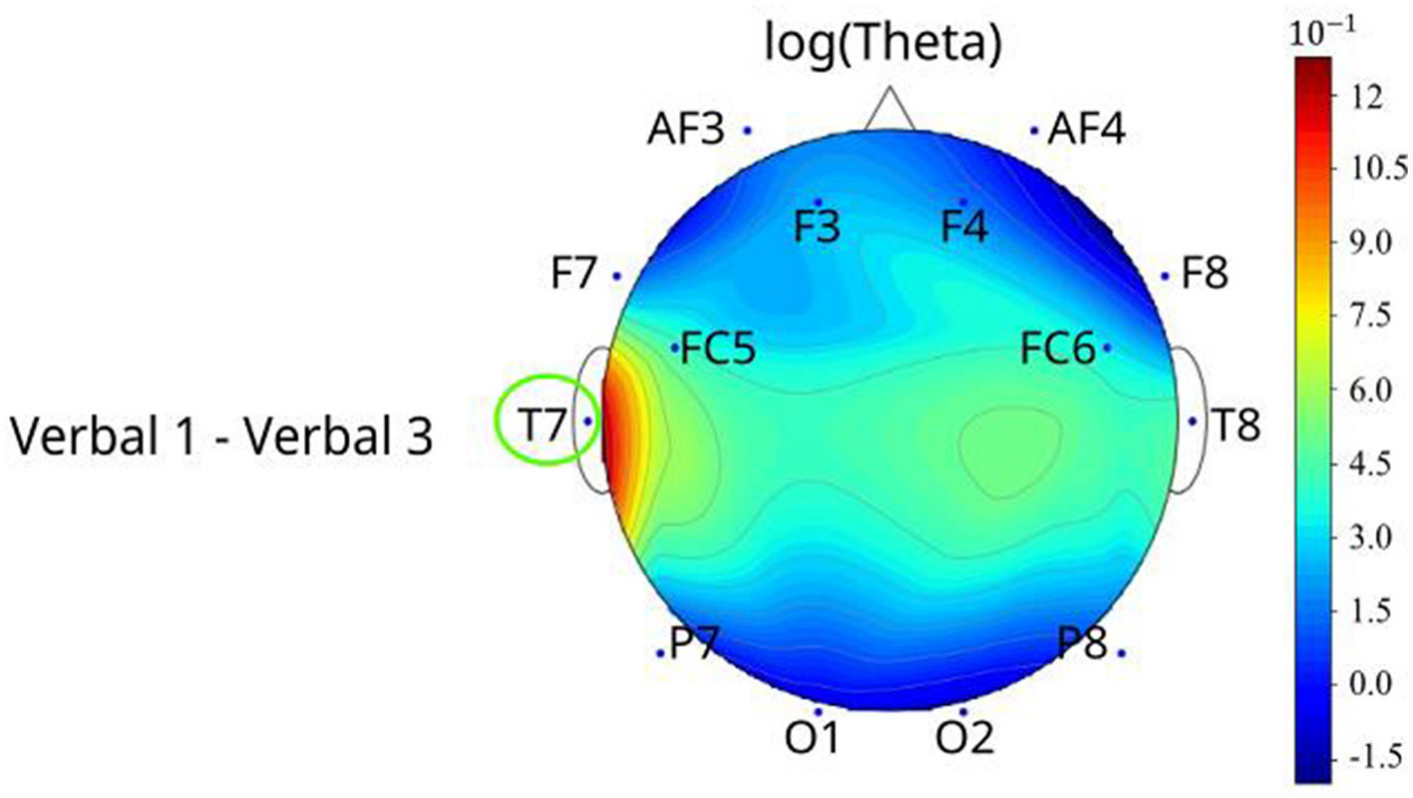
The topographical plots of the significant metric (after applying log transformation) among different verbal levels with channels that differed between pairwise comparisons circled. The topographical plots were made based on the value difference between two levels of log(metric).

For models of the verbal task (Figure 7), only log(theta) of T7 decreased (*p* = 0.0370) when the verbal levels increased in T7 (Level 1 was 1.45 higher than Level 2) (Figure 7). Appendix B.VII shows additional details on the verbal-based EEG metrics pairwise comparison results.

## 4 Discussion

For MWL induced by varying math levels, the performance metric number of questions answered emerged as the most effective. It was the only metric capable of distinguishing all MWL levels. However, another performance metric, the percentage of correct answers, was less effective, distinguishing only six out of ten level pairs. Notably, regardless of the models' granularity in detection, the levels identified as significantly different by performance metrics consistently showed a trend of decreasing performance as MWL levels increased. This observation aligns with findings reported in previous literature (Young et al., 2015). The self-reported OW scores effectively distinguished all math levels, with the exception of Levels 1&2. Similar to performance metrics, our findings corroborate earlier studies indicating that higher self-reported MWL levels are associated with increased MWL (Fallahi et al., 2016; Muñoz-de-Escalona et al., 2020).

With respect to eye movements, we found a longer average fixation duration, a fewer number of fixations and number of saccades, and a shorter average amplitude of saccades with higher MWL. This finding is reasonable, as high MWL would require more time to process complex information (Mahanama et al., 2022) and elicit greater engagement (Geisen, 2017), leading participants' attention to be more focused on task-relevant stimuli. The increased focus during more demanding tasks reduces the need for extensive visual search and exploration, limiting gaze shifts to a relatively small area relevant to the task. This results in fewer but more focused eye movements.

Another physiological sensor used in the study, the EEG, found that alpha bandpower decreased when MWL increased, a trend that aligns with findings from previous studies (Brookings et al., 1996; Fairclough and Venables, 2006; Jaquess et al., 2018). For example, memory retrieval and arithmetic tasks were shown to decrease alpha bandpower (Harmony et al., 1999). In contrast, previous studies observed that theta bandpower had a positive relationship with MWL, where high theta amplitude suggests sustained attention (Sasaki et al., 1996; Gevins et al., 1997). However, our present study indicates that theta bandpower was the highest at both Levels 1&2. In contrast, Level 3 had the lowest theta, while Levels 4&5 were in the middle. This contrasting finding may be partially explained by our simulation design. Specifically, participants were tasked to answer as many problems as possible continuously for 30 s at each milestone, regardless of the MWL level of the milestone. Therefore, participants' attention should have been sustained for the entire 30 s for all the MWL levels. While the overall tendency was for theta to decrease while MWL increased. This, combined with the behavioral results, suggests some disengagement may have occurred at the higher levels of difficulty. Disengagement at lower or higher demand levels is a common finding in previous literature (Dehais et al., 2020).

All of the metrics we investigated were able to discriminate between math demand levels, consistent with past work showing the utility of these metrics for detecting MWL. However, as with previous works, there appear to be some limitations in the granularity of the metrics (Zhou et al., 2022). Specifically, we found only one metric (number of questions answered) can distinguish five math levels completely. Although performance metrics are strongly associated with incremental changes in workload, there are significant limitations to the practical application of performance-based models in safety-critical jobs like SAR, where assistance or support should be provided prior to operators showing impaired performance. In addition, performance metrics in real-world settings may not be as easily measured or defined as the math and verbal tasks in this study. Instead, as the physiologically based models can be collected in real-time and physiological responses to workload are indirect and not task specific (Zhang et al., 2023), they may be more generalizable outside of a lab setting and may provide more opportunities for informing work and machine designs. Thus, relying solely on performance metrics is insufficient to represent MWL. The inclusion of subjective questionnaires and physiological data is also necessary for a more comprehensive understanding.

For all the other subjective and physiological metrics, math Levels 1&2 and math Levels 3&4 are the two most unlikely distinguishable level sets by all the metrics. This might be because the differences in demand required from math Levels 1&2 and 3&4 are less than the other level sets (for example, Levels 2&3), which makes these two sets harder to distinguish. Despite these challenges, we believe our presented models are a step toward our goal of predicting more granular levels of workload. Specifically, each pair was distinguished by one or more metrics, suggesting future work integrating or combining multiple metrics together can lead to the stated goal of a comprehensive granular workload prediction model.

Contrary to math models, only a limited number of metrics succeeded in differentiating between levels of verbal MWL. Specifically, no metrics distinguished verbal Level 2 from Level 1. A likely explanation for this observation was the required cognitive functions demanded by the verbal questions. Specifically, the verbal questions were hard for most participants regardless of MWL workload as they required deep reasoning on vocabulary pairings that many participants may not be familiar with (these questions were removed in United States SAT standardized testing in 2005). Except for the most challenging level, where participants struggled to answer questions correctly, the other levels may have seemed similar to them. In these levels, participants knew some answers and made educated guesses for the rest. This challenge may also be amplified by familiarity with the English language. However, although our sensing approach was not capable of distinguishing granular (high, medium, and low) levels of verbal MWL, several metrics did distinguish between Levels 1&3 and 2&3, which likely represent the large differences in MWL from surface to deep reasoning of the verbal questions.

Several opportunities for further research emerge from this study. Firstly, the effects of gender and other demographic variables were not considered, though some studies suggest that different genders may yield varying results in MWL measurement metrics (Hancock, 1988; Zeng et al., 2020). Furthermore, our participant pool was limited to a university population, potentially differing in psychological cognition from other societal groups. Expanding the participant demographic in future studies could provide more generalized insights. Another future work is the scope of the study in modulating MWL. The MWL demands were not tailored to specific scenarios like Search and Rescue (SAR) or human-robotic interaction. While this approach was intentional to understand MWL's fundamental mechanisms, future research should aim to replicate more complex, real-world demands. Moreover, in this study, we employed single-channel EEG analysis to observe bandpower trends in specific brain regions, as measured by 14 channels, despite the small sample size. However, multi-channel EEG analysis, which can provide deeper insights into complex cognitive tasks (like mental math and verbal tasks) that involve multiple brain regions, indicates the need for future studies to explore aggregate effects across channels. Additionally, the study's controlled laboratory setting limits the applicability of findings to real-world scenarios. Future research should investigate factors affecting sensor performance in naturalistic settings, such as motion artifacts impacting EEG readings (Kappel et al., 2017). Moreover, as has been demonstrated in past literature (Brookings et al., 1996; Jaquess et al., 2018), the relationship between MWL and physiological metrics is complex. Some metrics have a linear relationship, while others have a non-linear relationship. Our exploration of both linear and non-linear models indicates a context-dependent preference, highlighting the need for further research to clarify the nature of this relationship and its implications for MWL models in varied applications. Despite these limitations, our work demonstrates the potential of multi-modal sensing to distinguish granular levels of MWL in a simulated gaming environment.

## Data availability statement

The raw data supporting the conclusions of this article will be made available by the authors, without undue reservation.

## Ethics statement

The studies involving humans were approved by Institutional Review Board at Purdue University. The studies were conducted in accordance with the local legislation and institutional requirements. The participants provided their written informed consent to participate in this study. Written informed consent was obtained from the individual(s) for the publication of any potentially identifiable images or data included in this article.

## Author contributions

JW: Conceptualization, Data curation, Formal analysis, Investigation, Writing—original draft. CS: Conceptualization, Writing—review & editing. WB: Writing—review & editing. DY: Conceptualization, Supervision, Writing—review & editing.

## Footnotes

^1^https://cdn2.hubspot.net/hubfs/360031/official_SAT_practice_test_2002.pdf; https://cdn2.hubspot.net/hubfs/360031/official_SAT_practice_test_2001.pdf; https://cdn2.hubspot.net/hubfs/360031/official_SAT_practice_test_2003-04.pdf; https://cdn2.hubspot.net/hubfs/360031/official_SAT_practice_test_2004-05.pdf

^2^ https://www.tobiipro.com/product-listing/tobii-pro-glasses-2/

^3^ https://connect.tobii.com/s/article/Gaze-Filter-functions-and-effects?language=en_US#:~:text=Gaze%20Filter%20%2D%20I%2DVT%20(,pursuit%20and%20VOR%20eye%20movements

^4^ https://documentation.sas.com/doc/en/pgmsascdc/9.4_3.4/statug/statug_mixed_syntax01.htm
