# Supplementary material for: Granular estimation of user cognitive workload using multi-modal physiological sensors
Source: Front Neuroergon. 2024 Feb 27;5:1292627. doi: 10.3389/fnrgo.2024.1292627 (PMC10927958; doi:10.3389/fnrgo.2024.1292627)
Supplement: Supplementary file 1 [file Data_Sheet_1.docx]

Supplementary Material

# Supplementary Tables

**Appendix A**

Questions of Previous Experience with Video Games.

| Question | Options |
| --- | --- |
| 1. On a scale of 1 to 5, please rate your familiarity with video games. | 1-Not familiar at all; 5-Very familiar |
| 2. How frequent do you play video games? | 1-Never; 2-Occasionally; 3-Sometimes; 4-Often; 5-Always |
| 3. On a scale of 1 to 5, please rate how confidence you are when playing the video games? | 1-Not confident at all; 5-Very confident |

**Appendix B**

Significant Pairwise Comparisons Result

B.I.1 Significant Task Performance Result – Math-Based Models

| **Metric** | **(i) Demand Level** | **(j) Demand Level** | **Mean Difference (i-j)** | **P > \|t\|** |
| --- | --- | --- | --- | --- |
| Number of Questions Answered | Math 1 | Math 2 | 1.69 | 0.0001 |
|  |  | Math 3 | 4.13 | <.0001 |
|  |  | Math 4 | 5.14 | <.0001 |
|  |  | Math 5 | 6.43 | <.0001 |
|  | Math 2 | Math 3 | 2.44 | <.0001 |
|  |  | Math 4 | 3.45 | <.0001 |
|  |  | Math 5 | 4.74 | <.0001 |
|  | Math 3 | Math 4 | 1.01 | 0.0069 |
|  |  | Math 5 | 2.29 | <0.0001 |
|  | Math 4 | Math 5 | 1.29 | 0.0005 |
| Percentage of Correct Answers | Math 1 | Math 4 | 25.98 | 0.0013 |
|  |  | Math 5 | 51.26 | <.0001 |
|  | Math 2 | Math 4 | 26.27 | 0.0012 |
|  |  | Math 5 | 51.56 | <.0001 |
|  | Math 3 | Math 5 | 36.67 | <.0001 |
|  | Math 4 | Math 5 | 25.28 | 0.0015 |

B.I.2 Significant Task Performance Result – Math-Based Models (Square Root Transformation)

| **Metric** | **(i) Demand Level** | **(j) Demand Level** | **Mean Difference (i-j)** | **P > \|t\|** |
| --- | --- | --- | --- | --- |
| $\sqrt{Number of Questions Answered}$ | Math 1 | Math 2 | 0.28 | 0.0022 |
|  |  | Math 3 | 0.89 | <.0001 |
|  |  | Math 4 | 1.16 | <.0001 |
|  |  | Math 5 | 1.85 | <.0001 |
|  | Math 2 | Math 3 | 0.62 | <.0001 |
|  |  | Math 4 | 0.88 | <.0001 |
|  |  | Math 5 | 1.57 | <.0001 |
|  | Math 3 | Math 4 | 0.27 | 0.0028 |
|  |  | Math 5 | 0.95 | <0.0001 |
|  | Math 4 | Math 5 | 0.69 | 0.0005 |
| $\sqrt{Percentage of Correct Answers}$ | Math 1 | Math 4 | 0.25 | 0.0016 |
|  |  | Math 5 | 0.52 | <0.0001 |
|  | Math 2 | Math 3 | 0.16 | 0.0493 |
|  |  | Math 4 | 0.26 | 0.0009 |
|  |  | Math 5 | 0.54 | <0.0001 |
|  | Math 3 | Math 5 | 0.39 | <.0001 |
|  | Math 4 | Math 5 | 0.28 | 0.0004 |

B.I.3 Significant Task Performance Result – Math-Based Models (Exponential Transformation)

| **Metric** | **(i) Demand Level** | **(j) Demand Level** | **Mean Difference (i-j)** | **P > \|t\|** |
| --- | --- | --- | --- | --- |
| $e^{Number of Questions Answered}$ | Math 1 | Math 2 | 123029 | 0.0005 |
|  |  | Math 3 | 127274 | 0.0003 |
|  |  | Math 4 | 127199 | 0.0003 |
|  |  | Math 5 | 127769 | 0.0002 |
| $e^{Percentage of Correct Answers}$ | Math 1 | Math 4 | 0.44 | 0.0022 |
|  |  | Math 5 | 0.84 | <0.0001 |
|  | Math 2 | Math 4 | 0.43 | 0.0028 |
|  |  | Math 5 | 0.83 | <0.0001 |
|  | Math 3 | Math 5 | 0.14 | <0.0001 |
|  | Math 4 | Math 5 | 0.14 | 0.0050 |

B.I.4 Significant Task Performance Result – Math-Based Models (Logarithmic Transformation)

| **Metric** | **(i) Demand Level** | **(j) Demand Level** | **Mean Difference (i-j)** | **P > \|t\|** |
| --- | --- | --- | --- | --- |
| Log(Number of Questions  Answered ) | Math 1 | Math 3 | 0.81 | <0.0001 |
|  |  | Math 4 | 1.14 | <0.0001 |
|  |  | Math 5 | 1.59 | <0.0001 |
|  | Math 2 | Math 3 | 0.64 | <0.0001 |
|  |  | Math 4 | 0.97 | <0.0001 |
|  |  | Math 5 | 1.42 | <0.0001 |
|  | Math 3 | Math 4 | 0.33 | 0.0002 |
|  |  | Math 5 | 0.78 | <0.0001 |
|  | Math 4 | Math 5 | 0.45 | <0.0001 |

B.II.1 Significant Task Performance Result – Verbal-Based Models

| **Metric** | **(i) Demand Level** | **(j) Demand Level** | **Mean Difference (i-j)** | **P > \|t\|** |
| --- | --- | --- | --- | --- |
| Percentage of Correct Answers | Verbal 1 | Verbal 3 | 32.85 | 0.0002 |
|  | Verbal 2 | Verbal 3 | 18.57 | 0.0307 |

B.II.2 Significant Task Performance Result – Verbal-Based Models (Exponential Transformation)

| **Metric** | **(i) Demand Level** | **(j) Demand Level** | **Mean Difference (i-j)** | **P > \|t\|** |
| --- | --- | --- | --- | --- |
| $e^{Number of Questions Answered}$ | Verbal 1 | Verbal 3 | 0.50 | 0.010 |

B.II.3 Significant Task Performance Result – Verbal-Based Models (Logarithmic Transformation)

| **Metric** | **(i) Demand Level** | **(j) Demand Level** | **Mean Difference (i-j)** | **P > \|t\|** |
| --- | --- | --- | --- | --- |
| Log(Percentage of Correct Answers) | Verbal 1 | Verbal 3 | 5.29 | <0.0001 |
|  | Verbal 2 | Verbal 3 | 3.93 | 0.0010 |

B.III. Significant Subjective Questionnaire Result – Math-Based Models

| **Metric** | **(i) Demand Level** | **(j) Demand Level** | **Mean Difference (i-j)** | **P > \|t\|** |
| --- | --- | --- | --- | --- |
| Overall Mental Workload Score | Math 1 | Math 3 | -10.92 | 0.0009 |
|  |  | Math 4 | -18.47 | <.0001 |
|  |  | Math 5 | -28.98 | <.0001 |
|  | Math 2 | Math 3 | -7.52 | 0.0204 |
|  |  | Math 4 | -15.07 | <.0001 |
|  |  | Math 5 | -25.57 | <.0001 |
|  | Math 3 | Math 4 | -7.55 | 0.0209 |
|  |  | Math 5 | -18.05 | <0.0001 |
|  | Math 4 | Math 5 | -10.50 | 0.0012 |

B.IV. Significant Eye Tracking Metrics Result – Math-Based Models

| **Metric** | **(i) Demand Level** | **(j) Demand Level** | **Mean Difference (i-j)** | **P > \|t\|** |
| --- | --- | --- | --- | --- |
| Total Fixation Duration (ms) | Math 1 | Math 4 | 2497 | 0.0088 |
|  | Math 2 | Math 4 | 3450 | 0.0003 |
|  |  | Math 5 | 2604 | 0.0046 |
| Average Fixation Duration (ms) | Math 1 | Math 3 | -148 | 0.0178 |
|  |  | Math 5 | -342 | <.0001 |
|  | Math 2 | Math 5 | -261 | <.0001 |
|  | Math 3 | Math 5 | -195 | 0.0012 |
|  | Math 4 | Math 5 | -252 | <0.0001 |
| Number of Fixations | Math 1 | Math 3 | 7.13 | 0.0005 |
|  |  | Math 4 | 7.66 | 0.0017 |
|  |  | Math 5 | 11.16 | <.0001 |
|  | Math 2 | Math 3 | 5.09 | 0.0107 |
|  |  | Math 4 | 5.63 | 0.0051 |
|  |  | Math 5 | 9.13 | <.0001 |
|  | Math 3 | Math 5 | 4.04 | 0.0353 |
| Number of Saccades | Math 1 | Math 3 | 5.83 | 0.0018 |
|  |  | Math 4 | 7.14 | 0.0002 |
|  |  | Math 5 | 8.51 | <0.0001 |
|  | Math 2 | Math 3 | 4.51 | 0.0141 |
|  |  | Math 4 | 5.82 | 0.0017 |
|  |  | Math 5 | 7.19 | <0.0001 |
|  | Math 1 | Math 4 | 1.89 | 0.0161 |
| Average Amplitude of Saccades  (Degree) |  | Math 5 | 2.77 | 0.0003 |
|  | Math 2 | Math 5 | 1.92 | 0.0111 |
|  | Math 3 | Math 5 | 1.48 | 0.0460 |
| Total Amplitude of Saccades (Degree) | Math 1 | Math 3 | 111.76 | 0.0006 |
|  |  | Math 4 | 147.37 | <.0001 |
|  |  | Math 5 | 183.98 | <.0001 |
|  | Math 2 | Math 3 | 63.93 | 0.0439 |
|  |  | Math 4 | 99.54 | 0.0020 |
|  |  | Math 5 | 136.15 | <.0001 |
|  | Math 3 | Math 5 | 72.22 | 0.0188 |

B.V. Significant Eye Tracking Metrics Result – Verbal-Based Models

| **Metric** | **(i) Demand Level** | **(j) Demand Level** | **Mean Difference (i-j)** | **P > \|t\|** |
| --- | --- | --- | --- | --- |
| ${Average Peak Velocity of Saccade}^{2}$ | Verbal 1 | Verbal 3 | 9006.44 | 0.0003 |

B.VI. Significant EEG Metrics Result – Math-Based Models

| **Metric (Channel)** | **(i) Demand Level** | **(j) Demand Level** | **Mean Difference (i-j)** | **P > \|t\|** |
| --- | --- | --- | --- | --- |
| Log(alpha)  (F8) | Math 1 | Math 3 | 0.86 | 0.0002 |
|  |  | Math 4 | 0.48 | 0.0350 |
|  |  | Math 5 | 0.45 | 0.0367 |
|  | Math 2 | Math 3 | 0.61 | 0.0138 |
| Log(beta)  (T7) | Math 1 | Math 4 | 1.15 | 0.0067 |
|  | Math 2 | Math 4 | 0.94 | 0.0142 |
| Log(beta)  (F8) | Math 1 | Math 3 | 0.83 | <0.0001 |
|  |  | Math 4 | 0.54 | 0.0091 |
|  |  | Math 5 | 0.42 | 0.0312 |
|  | Math 2 | Math 3 | 0.44 | 0.0478 |
|  | Math 3 | Math 5 | -0.40 | 0.0375 |
| Log(theta)  (T7) | Math 1 | Math 3 | 1.00 | 0.0190 |
|  |  | Math 4 | 1.03 | 0.0144 |
|  | Math 2 | Math 4 | 0.76 | 0.0470 |
| Log(theta)  (T8) | Math 1 | Math 3 | 1.03 | 0.0040 |
|  | Math 2 | Math 3 | 0.95 | 0.0089 |
|  | Math 3 | Math 4 | -0.74 | 0.0428 |
|  |  | Math 5 | -0.73 | 0.0440 |
| Log(theta)  (F8) | Math 1 | Math 3 | 1.00 | 0.0003 |
|  |  | Math 4 | 0.55 | 0.0381 |
|  |  | Math 5 | 0.57 | 0.0275 |
|  | Math 2 | Math 3 | 0.78 | 0.0080 |

B.VII. Significant EEG Metrics Result – Verbal-Based Models

| **Metric** | **(i) Demand Level** | **(j) Demand Level** | **Mean Difference (i-j)** | **P > \|t\|** |
| --- | --- | --- | --- | --- |
| Log(theta)  (T7) | Verbal 1 | Verbal 2 | 1.45 | 0.0115 |
